# Pain Management and Functional Recovery after Pericapsular Nerve Group (PENG) Block for Total Hip Arthroplasty: A Prospective, Randomized, Double-Blinded Clinical Trial

**DOI:** 10.3390/jcm12154931

**Published:** 2023-07-27

**Authors:** Małgorzata Domagalska, Bahadir Ciftci, Tomasz Reysner, Jerzy Kolasiński, Katarzyna Wieczorowska-Tobis, Grzegorz Kowalski

**Affiliations:** 1 Department of Palliative Medicine, University of Medical Sciences, 61-245 Poznan, Poland; tomrey@wp.pl (T.R.); kwt@tobis.pl (K.W.-T.); gkowalski@ump.edu.pl (G.K.); 2 Department of Anesthesiology and Reanimation, Istanbul Medipol University, Istanbul 34214, Turkey; bciftci@medipol.edu.tr; 3 Kolasinski Clinic, Hair Clinic Poznan, 62-020 Swarzedz, Poland; colas@klinikakolasinski.pl

**Keywords:** total hip arthroplasty, PENG block, quality of life, pain management, regional anesthesia

## Abstract

Background: The immediate postoperative period after total hip arthroplasty can be associated with significant pain. Therefore, this study aimed to evaluate the effect of pericapsular nerve block on pain management and functional recovery after total hip arthroplasty. Methods: This prospective, randomized, double-blinded, placebo-controlled trial was conducted on 489 adult patients scheduled for total hip arthroplasty, ASA 1–2, operated under spinal analgesia. Participants were assigned to receive either a pericapsular nerve group (PENG) block with 20 mL of 0.5% ropivacaine or a sham block. Results: The primary outcome measure was the postoperative NRS score in motion. The secondary outcomes were cumulative opioid consumption, the time to the first opioid, and functional recovery. Demographic characteristics were similar in both groups. Intraoperative pain scores were significantly lower in patients who received the PENG block than in the control group (*p* < 0.0001). Also, the time to the first opioid was considerably longer in the PENG group (*p* < 0.0001). Additionally, 24% of PENG patients did not require opioids (*p* < 0.0001). Conclusions: The pericapsular nerve group showed significantly decreased opioid consumption and improved functional recovery. Pericapsular nerve group block improved pain management and postoperative functional recovery following total hip arthroplasty.

## 1. Introduction

Total hip arthroplasty is one of the most common major orthopedic interventions and improves patients’ quality of life and functional status [1]. However, despite these advantages, the immediate time after surgery can be associated with significant pain, which delays mobilization and increases the duration of hospitalization and the risk of thromboembolic events [2,3]. In total hip arthroplasty (THR), the pain is usually treated with an injection of a local anesthetic around the joint, known as “local infiltration analgesia”. Adequate pain management after total hip arthroplasty is critical for early rehabilitation and patient satisfaction. Moreover, the complex innervation of the hip joint makes a perfect regional anesthesia technique questionable. After surgery, regional anesthesia techniques for pain management include epidural analgesia, lumbar plexus block, parasacral block, fascia iliaca block, and femoral and obturator nerve block [2,4,5]. However, these procedures can lead to complications such as epidural hematoma, headache after surgery, or prolonged motor block with the subsequent prolonged hospital stay [6,7,8].

The obturator nerves, accessory obturator, and femoral nerve innervate the anterior hip capsule. The iliopubic eminence and inferomedial acetabulum were recommended as important bone landmarks to block the articular branches of these three nerves. Furthermore, the great role of the accessory obturator nerve and femoral nerve in the anterior hip innervation has also been stated.

LIA pursues the sensory nerve endings around the joints without decreasing the quadriceps strength. However, even with LIA, some patients experience pain in the days after total hip arthroplasty.

An international consensus of evidence-based experts recommends peripheral nerve blocks (PNB) as a central anesthetic method in THA to improve outcomes [9]. PNB for postoperative analgesia would also maintain quadriceps strength to facilitate early recovery. Common PNBs like a sciatic nerve block, femoral nerve block, lumbar plexus block, and fascia iliaca block cause quadriceps muscle weakness.

The pericapsular nerve group (PENG) block is an ultrasound-guided approach first described by Giron-Arango et al. [10]. The PENG block targets the articular branches of the obturator nerve, the accessory obturator nerve, and the femoral nerve, providing sensory innervation to the anterior capsule of the hip [7,11,12]. It has been used successfully in multimodal pain management for hip fractures [12,13] and for pain management after total hip arthroplasty [14]. It has been shown that the PENG block can protect the body, speeding up the first ambulance and recovery. However, some studies have shown that it can weaken the quadriceps, especially if the volume is more than 20 mL [15,16]. Therefore, we conducted a prospective, randomized, controlled, double-blinded trial to assess the effectiveness of a PENG block in improving analgesia and functional recovery following total hip arthroplasty. Our primary outcomes were the postoperative pain sores, and the secondary outcome measures included opioid consumption and functional recovery expressed by active elevation of the operated limb and walking by the balcony.

## 2. Patients and Methods

### 2.1. Study Design and Participants

This prospective, randomized trial was performed at the Independent Public Health Care Institution of the Ministry of the Interior and Administration in Poznań, Poland, in accordance with the Declaration of Helsinki. The Institutional Review Board of the Poznan University of Medical Sciences approved the study protocol on 17 June 2020, protocol number 496/20, and registered it at clinicaltrails.gov (NCT05944380). Written informed consent was obtained from all patients for this scientific contribution.

Enrollment was proposed before surgery for adults scheduled for elective primary unilateral total hip arthroplasty under spinal anesthesia, aged >18 years, and American Society of Anesthesiologists physical status 1 or 2.

Patients were not included in this study if they refused to participate, had a history of opioid abuse, had an infection of the site of needle puncture, were less than 18 years of age, were postponed as having ASA > 2, had an allergy to any of the drugs used in the study, had renal failure (estimated glomerular filtration rate of <15 mL/min/1.73 m^2^), liver failure, known or suspected coagulopathy, pre-existing anatomical or neurological disorders in the lower extremities, intellectual disability with problems in pain evaluation, and severe psychiatric illness.

### 2.2. Randomization

Patients were randomly allocated to receive ultrasound-guided PENG block, or sham block, by computer software using a 1:1 randomization list generated by the program nQuery Advisor (Statistical Solutions, Boston, MA, USA). The randomization lists were accessible to a researcher who was not involved in the study and concealed group assignments in consecutively numbered, sealed, opaque envelopes. A consultant anesthesiologist followed management to open the envelopes shortly before the nerve block performance to reveal the group allocation and perform the procedure according to the assignment. The patients, surgeons, operating room staff, and anesthesia team were masked from the study group allocation. Group blinding and unmasking occurred once the statistical analysis was complete.

All patients underwent primary total hip replacement (under spinal analgesia) performed by three surgical teams using the posterior approach at our tertiary institution.

The study subjects were subjected to at least 5 days of active follow-up. An independent researcher gathered the primary and secondary outcomes during in-person hospital visits.

### 2.3. Perioperative Management and Spinal Anesthesia Procedure

All the patients received standardized spinal anesthetic management as commonly practiced in our hospital. In both groups, the patients received 7.5 mg of midazolam p.o. and 8 mg of Dexamethasone i.v. half an hour before the procedure as a part of multimodal preemptive analgesia. For mild sedation, before the induction of anesthesia, intravenous doses of 2 mg of midazolam and 100 mg of fentanyl were given. In addition, all patients had spinal anesthesia, which was performed by injecting 20 mg of ropivacaine 0.5% through a 27G or 25G Whitacre needle at the L2–L3 or L3–L4 interspace with the patient sitting. Intravenous tranexamic acid 1000 mg and cefazolin 1 g were administered after spinal anesthesia and before surgery. There was no surgeon-delivered periarticular infiltration during the surgery.

### 2.4. PENG Block Procedure

**Figure 1:**
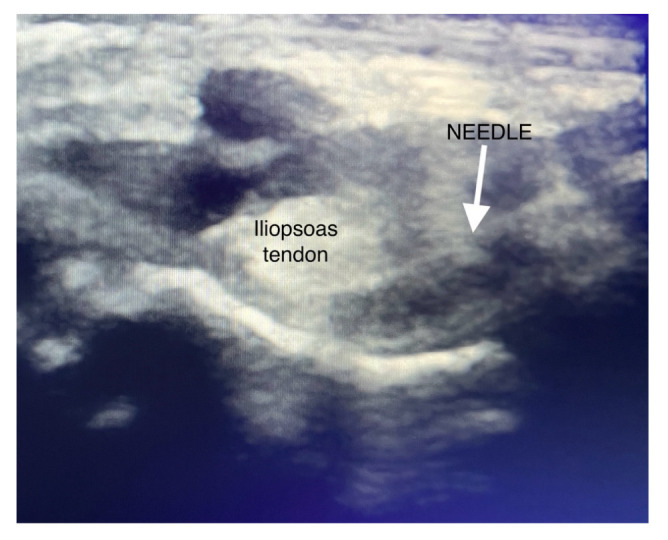
Injection technique of PENG block.

In both groups, the PENG block or sham block was performed after the spinal anesthesia and before the surgical incision, according to the technique described by Girón-Arango [10]. However, according to Peng et al. [17] and Tran et al. [18], we modified the original PENG block technique to avoid quadriceps weakness. A curvilinear probe (low frequency, 4–8 mHz) was used. The puncture was performed in the lateromedial direction, and the needle was placed more laterally, away from the surface of the iliopsoas tendon and between the anteroinferior iliac spine and the ilio-pubic eminence. After negative aspiration, 20 mL of 0.5% ropivacaine or 20 mL of 0.9% NaCl was injected laterally from the iliopsoas tendon, as seen in Figure 1. Three anesthesiologists performed the blocks. All had at least five years of experience of post-specialty clinical experience focused on regional anesthesia. During the surgery, basic hemodynamic parameters, opioid consumption (fentanyl), and operation time were measured.

### 2.5. Postoperative Analgesia Management and Evaluation of Outcomes

The patients were transferred to the post-anesthesia care unit (PACU) after the end of the surgery. In the PACU, the same postoperative multimodal analgesia was applied in both groups, which was consistent with acetaminophen 1 g i.v. every six hours, metamizole 1 g i.v. every six hours, and ketorolac 50 mg every twelve hours. The 5 mg morphine was administered if the NRS score was higher than 4 as rescue analgesia. When severe nausea or vomiting occurred, the patients were treated with 8 mg of Ondansentrone. All patients received thromboembolism prophylaxis daily with enoxaparin for 4 weeks postoperatively. Subsequently, after the first 10 postoperative hours, patients were ambulated with the help of the hiker.

### 2.6. Outcome Assessments

The primary outcome measures were pain scores at rest and during mobilization up to 5 days following surgery. At all postoperative time points (24, 48, 72, >72 h), patients were asked to rate perceived pain using an 11-point Numeric Pain Rating Scale (NRS: 0 indicating no pain and 10 indicating the worst pain imaginable) experienced at rest and during mobilization. The secondary outcomes included total opioid consumption and time to first opioid use obtained from the postoperative and orthopedic wards. Opioid consumption during 0–24, 24–48, and 48–72 h after surgery, and total opioid consumption at 72 h following surgery, were recorded. The consumption of the different types of postoperative opioid administration was converted to intravenous morphine equivalents. The functional recovery of each patient was tested by active elevation of the operated limb. The measurement was made 6 h postoperatively, and the ability to walk 3 steps by the balcony was assessed 10 h after surgery.

The outcome assessment was performed by a group of two clinicians (GK and KWT) who were blinded to the group allocation.

### 2.7. Statistical Analyses and Sample Size Calculation

To calculate the sample size, we studied our primary and secondary hypotheses that the PENG block improves postoperative analgesia. We estimated pain score density as a mean of 4 and SD of 6 based on the published data on total hip arthroplasty using PENG blocks [14]. We use a truncated Gaussian distribution with a range of 0 to 10, SD 6, and a mean of 4 for the PENG group to model the drift. Under these assumptions and two-sided = 5%, we simulated a sample of 234 patients in each group. With an overall sample size of 468 subjects, we estimated 95% power to detect differences in pain between groups as small as approximately 1. Statistical analysis was performed using GraphPad Prism 8 software (GraphPad Software Inc., San Diego, CA, USA).

The parametric distribution of numerical variables was evaluated using the Kołomogorov–Smirnov normality test. The differences between groups were analyzed by t-student or Mann–Whitney U test. Categorical variables were correlated with the Mann–Whitney U test, and an analysis of contingency was compared with Fisher’s exact test. Values are given as mean (standard deviation), median (interquartile range), or the number of patients (proportion). The balance of inpatient and operation idiosyncrasies between the randomized groups was determined by estimating the standardized difference, defined as the variation in proportions or means divided by the pooled standard deviation. Successively measured variables were postponed using a linear mixed model with the patient indicator as a random effect and group, time, and group-by-time interaction as fixed effects, adjusting for variables of patient and operation characteristics (sex, age, body mass index, ASA physical status, surgery duration, spinal anesthesia level). An unstructured covariance structure was applied. The Bonferroni correction was enforced to adapt for multiple comparisons. All analyses were accomplished using GraphPad Prism 8 software (GraphPad Software Inc., San Diego, CA, USA). A *p*-value of <0.05 was treated as statistically significant.

## 3. Results

### Patients and Operation Characteristics

**Figure 2:**
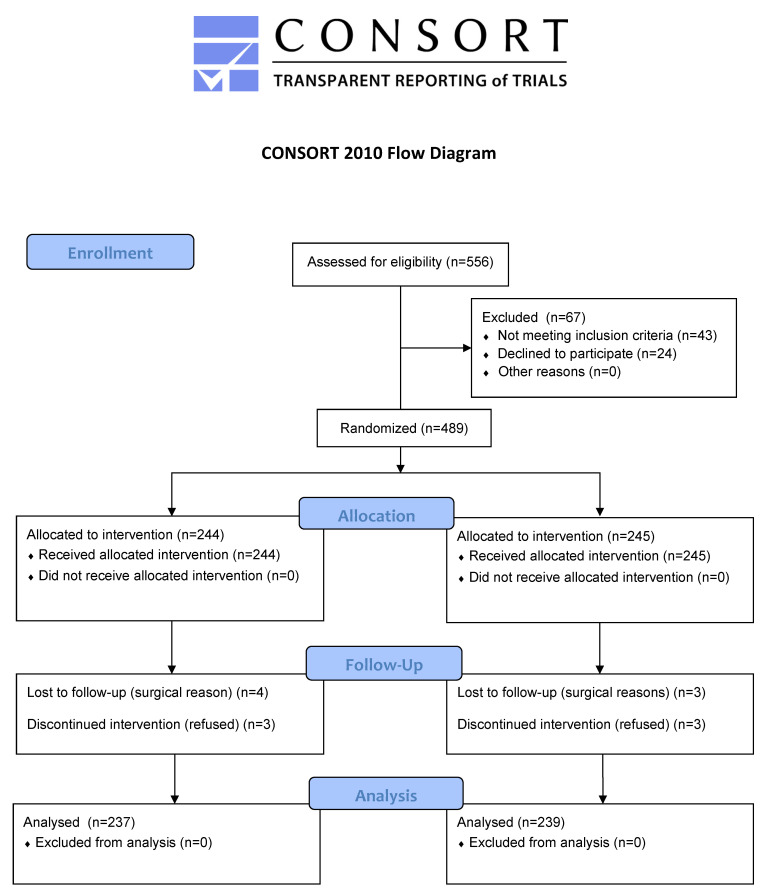
Consort Flow Chart.

Of 556 patients assessed for eligibility, 43 did not meet the inclusion criteria, and 24 preferred general anesthesia. The remaining 489 were randomly allocated between groups, as shown in Figure 2. No clinically relevant differences were apparent from group characteristics, as shown in Table 1.

**Figure 3:**
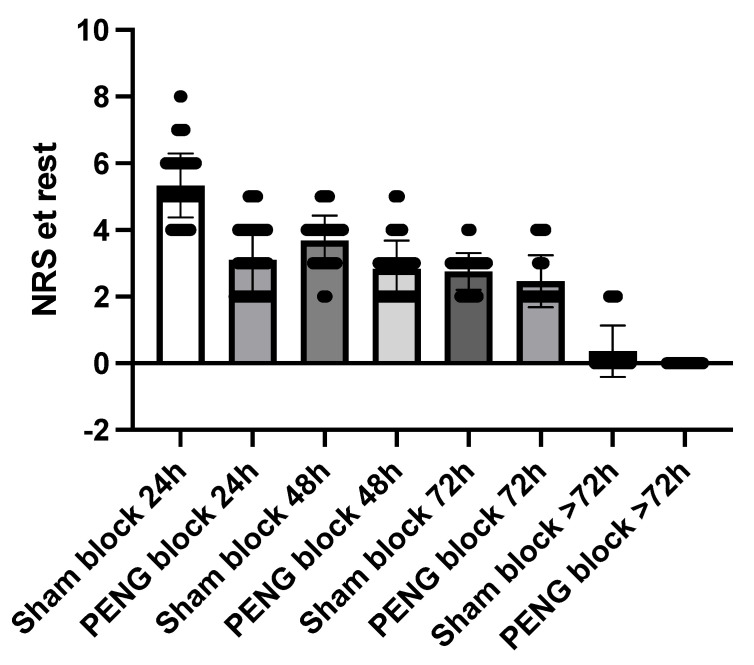
NRS at rest.

Postoperative pain scores are shown in Table 2 and Figure 2 and Figure 3. Patients who underwent the PENG block had lower NRS pain scores at rest at all time points. Comparing the PENG block to the sham block, NRS pain scores such as 3.1 vs. 5.3 at 24 h (*p* < 0.0001), 2.8 vs. 2.5 at 72 h (*p* < 0.0001), and 0.4 vs. 0 over 72 h (*p* = 0.0004), reveals better pain control.

**Figure 4:**
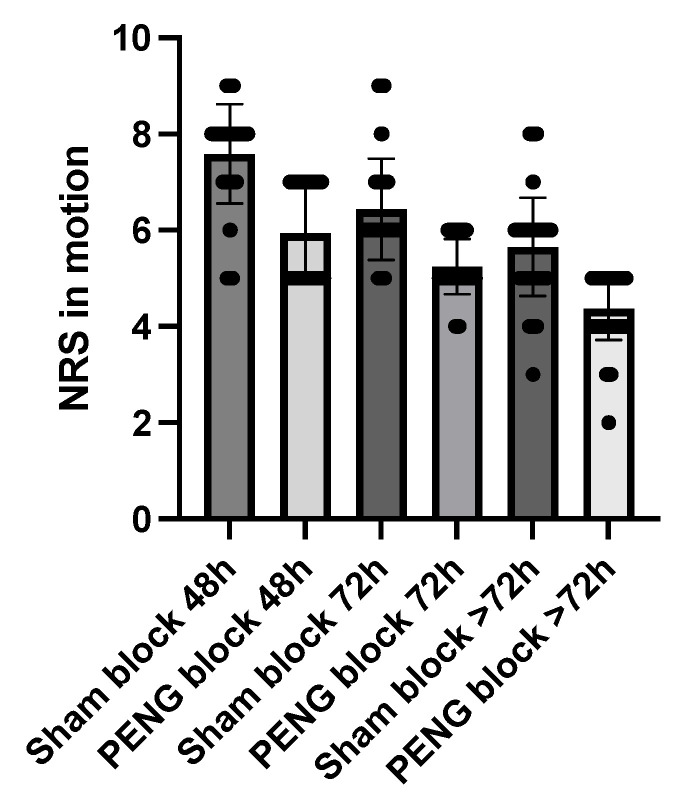
NRS et motion.

Also, NRS pain scores during movement were lower in the PENG block group at all time points, with a median of 5.9 vs. 7.6 at 48 h, 5.2 vs. 6.4 at 72 h, and 4.4 vs. 5.7 over 72 h, all *p* < 0.0001 (Figure 4).

**Figure 5:**
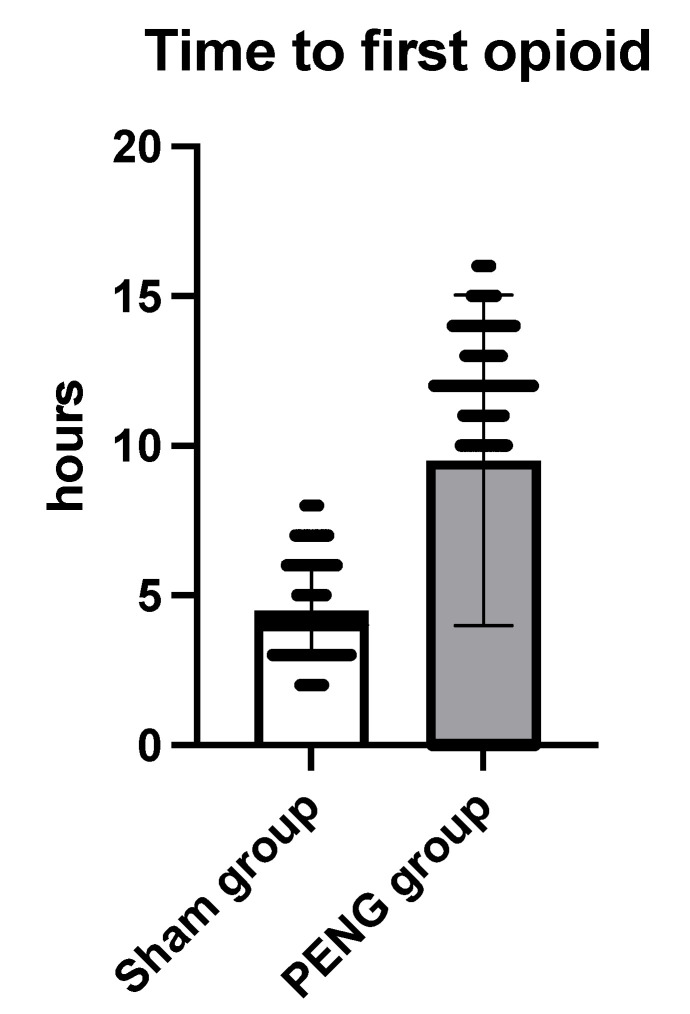
Time to first opioid.

Every patient in the Sham group received morphine intravenously for pain treatment. In contrast, 57 (24%) in the PENG group received none. As a result, the total opioid consumption, expressed in intravenous equivalents, was lower in the PENG group at all time points: 2.3 vs. 8.4 at 24 h, 0.5 vs. 2.5 at 48 h, and 0 vs. 0.3 at 72 h, all *p* < 0.0001. In addition, the mean time to the first opioid was 5 h shorter in the Sham group (*p* < 0.0001) (Figure 5). The results are shown in Table 2.

Quadriceps Strength in the operative leg measured 6 h after surgery by active elevation of the operated limb was higher in the PENG group. 127 (53%) patients in the PENG group could actively elevate the operated limb, compared to 25 (11%) patients in the Sham group. The remaining 47% of patients in the PENG group and 75% of patients in the Sham group could not actively elevate the operated limb due to accompanying pain.

Moreover, all patients in the PENG group could walk by the balcony 10 h after surgery, compared to 108 (47%) in the sham group, *p* < 0.0001. 53% of patients in the Sham group could not walk by the balcony due to accompanying pain.

## 4. Discussion

The major result of this study was that ultrasound-guided PENG block could improve pain relief after surgery in THA patients without weakening the quadriceps muscle. Additionally, patients in the PENG group took much longer until the first opioid, and half did not require opioids. Our results support the fact that the pericapsular nerve group significantly reduced pain scores during motion and opioid consumption. Moreover, it extended the time until the first opioid and improved functional recovery.

The PENG block is a relatively novel ultrasound-guided regional anesthesia technique designed to block the branches of the femoral, obturator, and obturator accessory nerves innervating the anterior capsule of the hip joint [10,14]. Currently, the PENG block is used for pain management in various hip surgeries, including fractures and hip replacements [11,12,13,14,17]. However, most recent evidence is limited to trials with small group sizes [11,14] and case reports [19,20,21,22]. Pascarella et al. [14], in their randomized, observer-masked, controlled trial, showed a significant reduction in opioid consumption, a shorter time to ambulation, and a better range of hip motion. Also, Lin et al. [16], in their double-blinded randomized comparative trial, showed that patients receiving PENG block for intraoperative and postoperative analgesia during hip fracture surgery experienced less postoperative pain with preserved quadriceps strength. Similar pain relief was also observed in our study. On the other hand, Zheng et al. [23] revealed that a PENG block added to intra-articular injection of local anesthetic provides a limited benefit to postoperative analgesia. However, Eti Korkusuz et al. [24] showed that ultrasound-guided PENG block offers better pain relief in treating hip osteoarthritis than the intra-articular injection of steroid-bupivacaine.

Quadriceps weakness was observed in some studies after the PENG block [16,25,26]. The exact mechanism of femoral nerve anesthesia after PENG block is controversial and results from local anesthetic spread via a plane between the psoas major and pectineus or intramuscularly [27]. According to Pascarella et al. [28] to avoid the short-term motor block, the needle tip should be placed medial to the iliopsoas eminence and under the iliopsoas tendon. Also, the clinician should observe a transversal spread with the tendon lifted during the injection. In our study, we placed the needle tip laterally and away from the undersurface of the iliopsoas tendon. Also, we reduced the volume of a local anesthetic to 20 mL to avoid motor weakness, as Çiftçi et al. [29] and Yeoh et al. [30] suggested in their studies. For this reason, we did not observe quadriceps weakness in our study. However, in our study, 47% of patients in the PENG group and 75% in the Sham group could not actively elevate the operated limb. The severe pain caused difficulty lifting the operated limb, similar to other studies [31,32].

A significant drawback of a single-shot PENG block is the limited time of analgesia. Also, rebound hyperalgesia after a single-shot nerve block has been reported [33,34]. Therefore, our study gave the patients acetaminophen, metamizole, and ketorolac to avoid rebound pain. Also, we decided to use systematic dexamethasone to prolong analgesia following a single-shot peripheral nerve block due to its proven effectiveness [35].

Only three researchers [10,19,36] reported that few patients experienced pain after the PENG block in the lateral femoral cutaneous nerve region. That is why we decided not to add the femoral cutaneous nerve block to the PENG block. In addition, we did not observe pain in the femoral cutaneous nerve region in our study. Furthermore, the pain scores were significantly lower in the PENG group, and 24% of patients did not need opioids, compared to 0% in the placebo group. Furthermore, the PENG block significantly reduced total opioid consumption and lengthened the time to first opioid consumption. Also, total opioid consumption was lower in the PENG group, and the patients in the PENG group did not require opioids 48 h postoperatively.

Our study suggests that the PENG block maintained optimal postoperative pain management, swift motor recovery, and lowered opioid consumption. Therefore, the PENG block may be a helpful anesthetic technique for postoperative pain control in modern, rapid hip surgery.

However, this study has limitations, such as the volume of the local anesthetic used for the PENG block, single-shot injection instead of the catheter, and the fact that we did not evaluate the dermatome levels. The functional recovery was assessed by active elevation of the operated limb 6 h after surgery and the ability to walk 3 steps by the balcony 10 h after surgery. Our study included only posterior total hip replacements, although this is the most commonly performed procedure worldwide.

## 5. Conclusions

The PENG block has proven to be a forceful opioid-sparing analgesic technique that boosts early postoperative mobilization and merits consideration as an effective analgesic option in total hip arthroplasty.

## Figures and Tables

**Table 1:** Baseline characteristics. Values are mean (SD) or number.

	Sham Block Group n = 237	Pericapsular Nerve Group (PENG) Block Group n = 239	*p* Value
ASA	2 (SD = 0.5)	2 (SD = 0.5)	0.8508
Age (years)	66 (SD = 5.1)	66 (SD = 5.8)	0.1001
Sex (F/M)	115/122	99/140	0.1429
BMI (kg/m^2^)	31 (SD = 2.9)	31 (SD = 3.2)	0.2108
NRS at rest—before surgery	4.1 (SD = 1.3)	4.3 (SD = 1.1)	0.2741
Spinal anesthesia needle level (L2/3 vs. L3/4)	31 (13%) vs. 206 (87%)	34 (14%) vs. 205 (86%)	0.7898
Surgery duration (min)	63 (SD = 7.6)	61 (SD = 8.3)	0.3211

ASA—American Society of Anesthesiologists Physical Status Classification System; F—female; M—male; BMI—body mass index; PENG—pericapsular nerve group.

**Table 2:** Study outcomes. Values are mean (SD) or numbers.

	Sham Block Group n = 237	Pericapsular Nerve Group (PENG) Block Group n = 239	*p* Value
**NRS postoperative**			
24 h	5.3 (SD = 1.0)	3.1 (SD = 1.0)	<0.0001
**NRS in motion**			
48 h	7.6 (SD = 1.0)	5.9 (SD = 1.0)	<0.0001
72 h	6.4 (SD = 1.1)	5.2 (SD = 0.6)	<0.0001
>72 h	5.7 (SD = 1.0)	4.4 (SD = 0.6)	<0.0001
**NRS at rest**			
48 h	3.7 (SD = 0.7)	2.8 (SD = 0.8)	<0.0001
72 h	2.8 (SD = 0.6)	2.5 (SD = 0.8)	<0.0001
>72 h	0.4 (SD = 0.8)	0	0.0004
**Postoperative opioid consumption**			
yes	237 (100%)	182 (76%)	<0.0001
no	0 (0%)	57 (24%)	
**Time to first opioid**			
hours	4.5 (SD = 1.6)	9.5 (SD = 5.5)	<0.0001
**Total opioid consumption** **(Intravenous morphine equivalents; mg)**			
0–24 h	8.4 (SD = 3.7)	2.3 (SD = 1.6)	<0.0001
24–48 h	2.5 (SD = 2.0)	0.5 (SD = 1.1)	<0.0001
48–72 h	0.3 (SD = 0.7)	0	<0.0001
**Functional recovery**			
Active elevation of operated limb	25 (11%)	127 (53%)	<0.0001
Walking by the balcony	108 (46%)	239 (100%)	<0.0001

NRS, numerical rating scale; mg, milligrams.

## Data Availability

The study datasets are available from the corresponding author at reasonable request.
